# The Increase of Diabetes

**Published:** 1891-11-07

**Authors:** 


### The Hospital

A Weekly Journal of

Science, Medicine, Nursing, and Philanthropy.

*For Hospitals, Asylums, Infirmaries, Dispensaries, Medical Practitioners, Students, Nurses, and the Charitable Public.*

Vol. XI.—No. 267.] [Nov. 7, 1891.

### THE INCREASE OF DIABETES.

The latest authoritative English work on diabetes affirms and proves certain facts which are of the first importance to those who live and work in large towns. It is to be noted that in Great Britain the population is rapidly concentrating itself in such places. One of the most urgent of the obligations that are imposed upon scientific medical men by their possession of knowledge is the imperative duty of imparting to the public at large that kind of medical knowledge of which it can make daily use. We do not believe for a moment that the average man when sick is capable of treating himself medically with intelligence any more than we believe him to be capable of making his own coats and trousers. "Every man to his trade" is one of those propositions the reasonableness of which is so plainly obvious that nobody but a fool would waste time in proving it. But scientific medical men and experimental physiologists have made certain discoveries of late years which, though brought to light in connection with medicine, ought to be in no sense the peculiar property of medicine. They belong, like morality and atmospheric air, to everyone who has the capacity for laying hold of them.

Diabetes is a disease of man rather than of animals, of the intellectual man rather than of the dull and stupid, and of the town man rather than of the inhabitant of the country. It is a disease which ought to be practically non-existent, and which would be nothing more than a rare curiosity if every man had a reasonable mind and fairly prosperous circumstances. The disease is one of those effects which serve as indicators of the general utility of the science of the times and of the practical advance of the human race. Where there is much diabetes we know there is excessive competition and strain among men, and much failure and premature closing of careers. Dr. Saundby informed the College of Physicians not long ago that the contrast between the number of diabetics in towns and those in country districts was very striking. He affirmed also that the proportion of those attacked by the malady increases from year to year; he further showed that among the cultured and grain-eating inhabitants of India, as well as among the Jews and Turks, the affection was remarkably prevalent. All these facts indicate with unmistakable clearness the class of causes that give rise to diabetes; and those causes we know to be, for the most part, preventible. The world needs to be instructed in these matters. It is an absolutely surprising thing that physicians, like Dr. Saundby, for example, never seem to think of the duty they owe to the public to impart their special knowledge. They teach the few who know as well as themselves, and leave the many to struggle in darkness, without showing them a single ray of light.

Among the most distinctly marked causes of diabetes are mental strain, worry, and sleeplessness. How are we to prevent men overworking themselves, worrying, and falling into sleeplessness? The question is often asked in the most pessimistic of moods. We must prevent them exactly as we prevent the too greedy man from stealing, by impressing upon them a rational and wholesome fear of the consequences. The least instructed of town doctors knows very well that over mental strain, worry, and sleeplessness, tend to produce diabetes; but the most intelligent of laymen knows nothing at all about the subject. Men work too hard and too long, and they fret and worry themselves excessively in order that they may get rich, or that they may live at a social level higher than that to which they have been accustomed. No practical man would lay it down as a principle of action that men are to take life easy and altogether avoid strenuous effort for the sake of keeping their health and living long. The best races of men are those who strive most to better their condition. But in every contest there are certain "rules of the game." It is imperative, not only to know the rules of the game, but to observe them, if victory is to be secured. The rules of the game are generally difficult in proportion to the importance of the contest and the duration of time over which it extends. Now, is there any contest in life of equal importance to the game of life itself? All sorts of people, from Confucius and Moses downwards, have tried their hands at teaching men the principles and details of the art of living. The fault of most of them has been that they have neglected the material part of man. They have striven to polish the jewel of the soul and despised the casket of the body. But recent generations have discovered the unlooked-for fact that the soul and the body are so intimately connected that they cannot be separated. The neglect of the body is, therefore, the neglect of the soul; and the overtraining and stimulation of the soul results in the exhaustion and wearing out of the body.

It is now as it always has been—men perish for lack of knowledge. The most urgent need of the men and women of this generation is the need of knowledge in the art of making the right use of themselves; the art of so using themselves as to bring out the best that is in them, and yet to avoid seriously injuring or destroying themselves. There are many fallacies abroad in the world on these points. The saintly man aspires "to wind himself too high, for mortal man beneath the sky"; and if he be a preacher he continually worries other people to persuade them to ascend with him the same impossible heights. The scientific man, not content with much that is true and a little that is new, labours to wring from nature ever more and more of the merely new, and like the lark striving to scale the skies when imprisoned in a cage, beats out his brains in the attempt. Similarly the politician, forced forward by the eager struggling of the multitude, is for ever racking his brains to add new planks to his political platform, and exhausts both his intellect and his emotions in the interminable task. A better knowledge of the rules of the game would put a check upon these unnatural activities. The knowledge that is wanted is that which the observant physician acquires in his consulting-room.

The doctor is like a judicial spectator at the tournament of life. He often knows years beforehand who will go down in the struggle, and who will survive to be a hoary veteran. If he had his way he would frequently withdraw men from the battle for a little time and give them a period of rest. Others he would move from the central fury of the fight to posts of less stress and danger; often he would raise aloft a white flag of truce and give all the combatants a period of repose and quiet. The physician would do these things if he could, but he cannot. All that he can do in his consulting-room is to take charge of the wounded, and restore them to health and bravery. But undoubtedly the greater function of teaching and judging awaits the scientific medical man. The mantles of the prophet and the schoolmaster are being prepared for him. He must teach the fighters as well as tend the wounded. This is the call of the modern world to the medical man. Woe be to him if fear, or mental blindness, or any other thing prevent him obeying the call.

The physician of the present day urges men to abate the ardour of the conflict. He tells them that if they would but give fifteen years to the work they strive to do in ten, they would not only do the work very much better, but they would preserve health and gain happiness. He who crams the work of fifteen years into ten, often spoils the work and almost invariably ruins himself. Most men think they will be happy when they have finished their work; the wise man finds his happiness in the work itself. The work of the present day demands, above all things, coolness and patience; the labours of the day should be dropped absolutely as soon as they are over; they should be never carried home. The evening should be given to recreation, the night to sleep. When, as sometimes happens, there is a period of increased stress and strain, it should always be followed by a period of change and rest. Drugs have but a small part to play in the working world. In the case of diabetes there is no one remedy which is of more than temporary and strictly limited service. The habit of health, of easy mindedness, of contentment, of indifference to the ways of other men, this alone is of real avail in the case of those who are threatened with diabetes. It is so all the way round. We must have soberer ambitions and quieter ways. If we do not, diabetes and other like maladies will increase upon us more and more. The danger threatens not only individuals and families, but the whole population. We ought to pray for statesmen, physicians, and teachers of all kinds with cool heads and steady and unyielding tempers. The example and the influence of leaders of this kind is salvation both to families and the State.

